# Safety and efficacy of salvage reirradiation for recurrent high-grade gliomas: a retrospective analysis

**DOI:** 10.1007/s11604-025-01941-z

**Published:** 2026-01-05

**Authors:** Miki Tsujii, Yaichiro Hashimoto, Yuka Kaizu, Kenta Ohmatsu, Sawa Kono, Shigehiko Kuribayashi

**Affiliations:** Department of Radiation Oncology, Tokyo Women’s Medical University School of Medicine, 8-1, Kawada-cho, Shinjuku-ku, Tokyo, 162-8666 Japan

**Keywords:** High-grade gliomas, Glioblastoma, Radiotherapy, Recurrent, IMRT

## Abstract

**Purpose:**

To assess the feasibility, safety, and clinical outcomes of salvage reirradiation, using intensity-modulated radiotherapy (IMRT) with a total dose of 30 Gy and cumulative equivalent dose in 2 Gy fractions (EQD2) limited to ≤ 96 Gy in patients with recurrent high-grade gliomas.

**Materials and methods:**

This retrospective study included 24 patients with recurrent high-grade gliomas who underwent IMRT-based reirradiation between 2014 and 2021. Treatment planning was performed using computed tomography-magnetic resonance imaging fusion images, and radiotherapy was delivered via IMRT or volumetric-modulated arc therapy. No concurrent chemotherapy was administered, and the cumulative EQD2 to the brain was restricted to 96 Gy.

**Results:**

The median follow-up was 8 months (range, 1–31 months). The median overall survival was 8 months, with 1- and 2-year overall survival rates of 29% and 4%, respectively. No grade ≥ 2 toxicity or cases of radiation necrosis were observed. All patients completed the planned treatment course without interruption.

**Conclusion:**

Salvage reirradiation using IMRT with an EQD2 limited to ≤ 96 Gy was a feasible and well-tolerated treatment strategy for select patients with recurrent high-grade gliomas. This approach may offer a modest survival benefit with minimal toxicity and warrants further investigation in prospective trials.

## Introduction

High-grade gliomas (HGGs), particularly glioblastoma (GBM), are the most common and aggressive primary brain malignancies in adults. GBM is characterized by a nearly universal recurrence rate, despite standard treatment such as maximal surgical resection followed by chemoradiotherapy with temozolomide [1, 2]. Recurrence typically occurs within the previously irradiated field, reflecting the tumor’s infiltrative growth pattern and resistance to conventional therapies [3]. Given the limited efficacy of salvage surgery and chemotherapy in the recurrent setting, salvage radiotherapy (i.e., reirradiation) has gained increasing attention as a strategy to improve local control and prolong survival. Advances in radiotherapy techniques, such as intensity-modulated radiotherapy (IMRT) and volumetric-modulated arc therapy (VMAT), have enabled highly conformal dose delivery while minimizing exposure to surrounding normal tissue, thereby making reirradiation a more feasible and safer option [4, 5]. Reirradiation is consequently emerging as a promising component of multimodal salvage therapy for recurrent malignant gliomas.

Growing evidence supports the efficacy and safety of salvage radiotherapy in patients with recurrent GBM; however, several essential uncertainties remain. The optimal total dose, fractionation schedule, and target volume delineation for reirradiation remain under debate, partly owing to substantial heterogeneity among published studies in treatment protocols and patient selection criteria [6, 7]. Hypofractionated stereotactic radiotherapy has demonstrated acceptable local control and survival benefits with limited toxicity in selected patients [8, 9]; however, most data are derived from retrospective series or small prospective trials. Moreover, emerging data suggest that extended radiation fields and high cumulative doses may exacerbate lymphopenia, potentially reducing the effectiveness of immunotherapies [2]. Therefore, there is an urgent need to refine reirradiation strategies that balance local tumor control with immune preservation and toxicity minimization.

In response to these clinical needs, in the present study, we retrospectively evaluated the safety and efficacy of salvage reirradiation using IMRT for patients with recurrent HGGs. In particular, treatment outcomes, toxicity profiles, and overall survival were reported after a standardized protocol of 30 Gy in 10 fractions, with cumulative equivalent dose in 2 Gy fractions (EQD2) restricted to ≤ 96 Gy to mitigate the risk of radiation necrosis. Through this institutional experience, we aim to contribute to the development of optimized, safe reirradiation strategies for this challenging patient population.

## Materials and methods

### Patients

This retrospective study included 24 patients with recurrent HGGs who underwent salvage radiotherapy at our institution between April 2014 and December 2021. The eligibility criteria included a histopathological diagnosis of glioblastoma, anaplastic astrocytoma, anaplastic oligodendroglioma, or anaplastic oligoastrocytoma, based on the 2007 or 2016 World Health Organization classification. Patients were excluded if the interval between the initial radiotherapy and reirradiation was < 6 months or if they declined salvage treatment. All patients had previously received standard initial therapy comprising surgery and radiotherapy with or without temozolomide chemotherapy. Tumor recurrence was confirmed via magnetic resonance imaging (MRI) in conjunction with clinical deterioration. A multidisciplinary tumor board decided to proceed with reirradiation. Clinical data, which included age, performance status, histological subtype at recurrence, and previous treatments, were retrospectively collected from electronic medical records. The study design was approved by our institution’s Ethics Review Board (protocol number: 2023-0179), and informed consent was obtained from all participants for this treatment.

### Radiotherapy

**Fig. 1:**
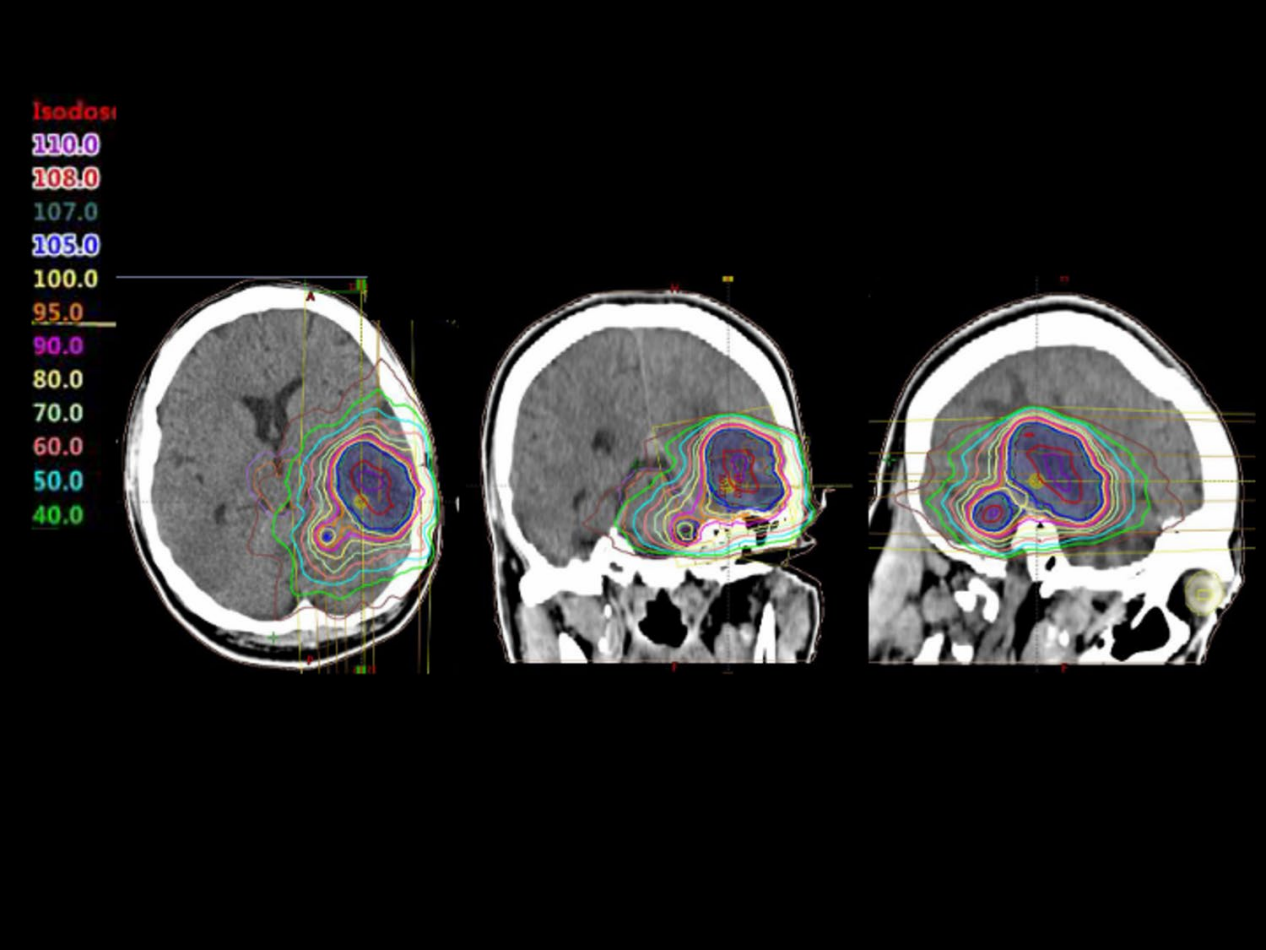
Representative dose distribution of salvage reirradiation using intensity-modulated radiotherapy. The axial plane illustrates the planning target volume (PTV) in red and the 95% isodose line in yellow. The dose distribution shows high conformality and appropriate sparing of surrounding normal brain tissue

All patients received salvage radiotherapy using VMAT, an arc-based implementation of IMRT, delivered via linear accelerator-based systems. For simplicity, these treatments were collectively referred to as IMRT in this study. Treatment planning was based on non-contrast-enhanced computed tomography (CT) images, which were co-registered with contrast-enhanced MRI to enhance target delineation accuracy. Patients were immobilized using a thermoplastic mask, and image-guided radiotherapy with daily cone-beam CT was performed to ensure accurate treatment delivery. The gross tumor volume (GTV) was defined as a contrast-enhancing recurrent lesion on MRI. A planning target volume (PTV) was generated by expanding the GTV by 2–5 mm, depending on anatomical considerations. A total dose of 30 Gy was delivered in 10 daily fractions of 3 Gy over 2 weeks. The cumulative biological dose, incorporating both initial and salvage radiotherapy, was calculated as the EQD2 using the linear-quadratic model with an α/β ratio of 10. The cumulative EQD2 to the brain was limited to ≤ 96 Gy to minimize the risk of radiation necrosis. No concurrent chemotherapy was administered during the salvage radiotherapy course. A representative dose distribution for IMRT-based salvage reirradiation is shown in Fig. 1.

### Follow-up and outcome measures

Patients were monitored regularly via clinical examinations and brain MRI scans. The median follow-up was 8 months (range, 1–31 months). The primary endpoint was overall survival (OS), defined as the interval from the start of salvage radiotherapy to the date of death from any cause or the last follow-up visit. Toxicity was assessed using the Common Terminology Criteria for Adverse Events, version 5.0 (National Cancer Institute, Bethesda, MD, USA; 2017). Radiographic responses and potential radiation-induced changes, including necrosis, were evaluated on follow-up MRI by experienced neuro-oncologists and radiation oncologists. In patients in whom new or enlarged contrast-enhancing lesions were observed after treatment, clinical correlation and serial imaging were used to distinguish actual progression from treatment-related changes.

### Statistical analysis

Statistical analyses were performed using Microsoft Excel for Mac, version 16.26 (Microsoft Corporation, Redmond, WA, USA). OS was estimated using the Kaplan–Meier method, and the 1- and 2-year OS rates were calculated. Given the retrospective nature and limited sample size of this study, only descriptive statistics were reported, and no formal hypothesis testing was conducted. Categorical variables were summarized as frequencies and percentages, and continuous variables were presented as the median and range. A *p-*value < 0.05 indicated a statistical significance. Multivariate analysis was performed using the Cox proportional hazards regression model in an exploratory manner to identify potential prognostic factors, recognizing the limited statistical power owing to the small sample size.

## Results

### Patients’ characteristics

Twenty-four patients with recurrent HGGs were included in the analysis. Table 1 presents the characteristics of all the patients and their tumors. The median age at reirradiation was 49 years (range, 22–70 years), and 13 (54%) patients were male. The Karnofsky Performance Status score at the time of recurrence was ≥ 80 in 11 (46%) patients, 60–70 in 12 (50%) patients, and 30 in 1 (4%) patient. Histological diagnoses at recurrence included glioblastoma in 10 (42%) patients, secondary glioblastoma in 3 (13%) patients, anaplastic astrocytoma in 8 (33%) patients, anaplastic oligoastrocytoma in 1 (4%) patient, and anaplastic oligodendroglioma in 2 (8%) patients.

**Table 1:** Baseline characteristics of patients with recurrent high-grade gliomas

	Overall cohort	
	(n = 24)	
Median age at recurrence (min/max), [y]	49	22/70
Median time from first RT to reirradiaion (min/max), [m]	17	5/118

	n	%
Gender		
Male	13	54
Female	11	46
KPS at recurrence		
80–100	11	46
60–70	12	50
30–50(30)	1	4
Histology at recurrence		
Recurrent glioblastoma	10	42
Secondary glioblastoma	3	13
Recurrent astrocytoma grade 3	8	33
Recurrent oligoastrocytoma grade 3	1	4
Recurrent oligodendroglioma grade 3	2	8
First Radiation		
60 Gy	22	92
50 Gy	2	8
Recurrence pattern		
In-field	17	71
Marginal	2	8
Out-field	5	21
Chemotherapy at recurrence		
Temozolomide	2	8
Bevacizumab	1	4
None	21	88

Demographic and clinical features at the time of salvage reirradiation include age, performance status, histologic subtype, recurrence pattern, interval since initial radiation, and concurrent therapies

All patients had previously received radiotherapy, with a dose of 60 Gy administered to 22 (92%) patients. The median interval between the initial radiotherapy and reirradiation was 17 months (range, 5–118 months). With regard to the recurrence pattern, 17 (71%) patients experienced in-field recurrences, 2 (8%) patients had marginal recurrences, and 5 (21%) patients had out-of-field recurrences. At the time of reirradiation, 21 (88%) patients did not receive concurrent chemotherapy, whereas 2 (8%) patients received temozolomide and 1 patient (4%) received bevacizumab.

### Survival outcomes

**Fig. 2:**
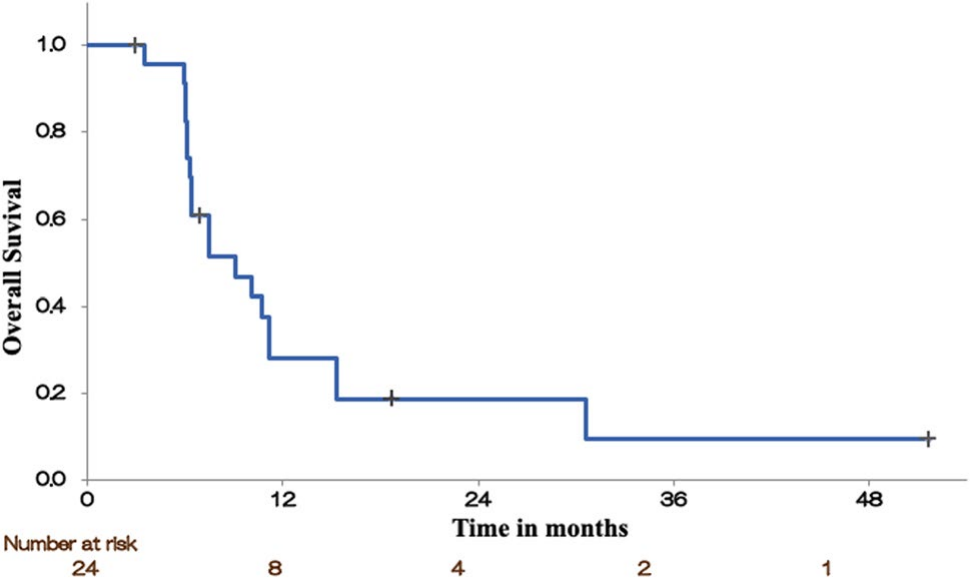
Kaplan–Meier curve for overall survival after salvage IMRT. The median overall survival was 8 months. The 1- and 2-year overall survival rates were 29% and 4%, respectively. Censoring events are indicated using tick marks on the survival curves. *IMRT* intensity-modulated radiotherapy

The median follow-up duration after salvage radiotherapy was 8 months (range, 1–31 months). The median OS from the start of reirradiation was 8 months. The 1- and 2-year OS rates were 29% and 4%, respectively. The Kaplan–Meier survival curve is shown in Fig. 2.

Multivariate Cox regression analysis identified glioblastoma histology as an independent predictor of a poorer OS (hazard ratio [HR], 2.41; 95% confidence interval [CI], 1.17–5.18; *P* = 0.02), after adjusting for age, isocitrate dehydrogenase (IDH) mutation status, extent of resection, and concurrent temozolomide use (Table 2). Consistent with this finding, the survival curves showed that patients with glioblastoma had notably poorer outcomes than those with anaplastic astrocytoma and other histologies.

**Table 2:** Multivariate Cox regression analysis for overall survival

Variable	HR	96% CI	*p*-value
Age (per year)	1.03	0.99–1.07	0.11
Male (vs Female)	1.25	0.72–2.14	0.42
GBM (vs AA)	2.41	1.17–5.18	**0.02**
IDH mutation (+ vs −)	0.58	0.31–1.09	0.09
RT to reirradiation	0.98	0.92–1.04	0.29
TMZ use	1.12	0.61–2.04	0.72

Bold values indicate statistical significance (p 0.05)

Hazard ratios (HRs), 95% confidence intervals (CIs), and *p*-values for variables, which include age, IDH mutation status, extent of resection, concurrent temozolomide use, and GBM histology

*GBM* glioblastoma, *IDH* isocitrate dehydrogenase, *RT* radiotherapy, *TMZ* temozolomide

### Toxicity

No grade ≥ 2 acute or late toxicity was observed during or after salvage radiotherapy. All patients completed the prescribed 30 Gy in 10 fractions without treatment interruption. Follow-up MRI revealed no cases of radiographically or clinically confirmed radiation necrosis.

## Discussion

In this retrospective study, we demonstrated the feasibility and safety of salvage radiotherapy using IMRT with a total dose of 30 Gy in 10 fractions for patients with recurrent HGGs. The median OS was 8 months, with 1- and 2-year OS rates of 29% and 4%, respectively. An important finding was that no grade ≥ 2 toxicity or cases of radiation necrosis were observed, suggesting that our protocol—which employed tight PTV margins and a cumulative EQD2 constraint of ≤ 96 Gy—was well-tolerated. In our protocol, the cumulative EQD2 was limited to ≤ 96 Gy, calculated using an α/β ratio of 10. Late toxicities such as radiation necrosis are more appropriately evaluated with an α/β ratio of 2; therefore, this threshold corresponds to approximately 97.5 Gy EQD2(α/β = 2). This cumulative dose level remains within the tolerance ranges reported in previous studies, supporting the safety of our reirradiation strategy [5].

These findings support the potential role of IMRT-based reirradiation as a salvage treatment in appropriately selected patients.

Our results are also consistent with those of prior series on reirradiation for recurrent HGGs. Several studies have shown the feasibility of hypofractionated stereotactic regimens [8, 9], while other retrospective cohorts and reviews have reported similar survival outcomes [4, 6, 7]. Furthermore, emerging concepts such as immune-preserving strategies in radiotherapy planning for GBM [5] highlight the importance of balancing efficacy and safety, and our approach may provide a framework for such integration. The favorable toxicity profile observed in our cohort may be attributed to meticulous treatment planning, including the use of CT–MRI fusion imaging for accurate target delineation, and the application of small PTV margins (2–5 mm) to reduce unnecessary dose to the surrounding brain tissue. Previous studies have shown that cumulative doses exceeding 100 Gy EQD2 are associated with increased risks of necrosis and neurologic toxicity, particularly in the setting of overlapping radiation fields [5]. By contrast, our protocol maintained conformity and spared the adjacent normal tissue, which was consistent with emerging evidence supporting the safety of hypofractionated reirradiation under strict dose constraints [4].

The survival outcomes in our cohort were consistent with those of previous reports on salvage radiotherapy for recurrent HGGs, in which the median OS typically ranges 5–12 months [6, 8]. Notably, most (88%) of our patients did not receive concurrent chemotherapy during reirradiation but did achieve comparable outcomes. This finding suggested that radiotherapy alone may contribute meaningfully to disease control and symptom palliation, particularly in patients with preserved performance status. Furthermore, multivariate analysis revealed that GBM histology was independently associated with poorer survival, underscoring the prognostic impact of tumor biology on treatment outcomes. However, given the limited sample size, this multivariate Cox regression should be regarded as exploratory and interpreted with caution.

IMRT-based reirradiation may serve as a valuable component of multidisciplinary care, particularly for patients who are ineligible for repeat surgery or further systemic therapy because of previous toxicity or poor tolerance. As systemic options such as immunotherapy gain relevance in neuro-oncology, the ability to deliver localized radiation with minimal lymphotoxicity assumes increasing importance. Previous studies [2] have shown that extensive radiation volumes and high cumulative doses can exacerbate lymphopenia, thereby potentially impairing immune responses. The dosimetric strategy adopted in this study may provide a valuable model for future integration with immunomodulatory therapies.

This study had several limitations. Its retrospective design and small sample size limit the generalizability of the results. In addition, the small sample size limited the ability to perform fully adjusted multivariate analyses, and some clinically relevant covariates could not be adequately controlled for. The absence of molecular biomarker data, such as O6-methylguanine-DNA methyltransferase promoter methylation or IDH mutation status, precluded the analysis of their prognostic or predictive value. Given the well-established prognostic significance of these biomarkers in gliomas, their absence represents a major limitation of this study. Moreover, toxicity assessment in this study was limited to clinical adverse events graded using the Common Terminology Criteria for Adverse Events. Neurocognitive function and quality-of-life outcomes were not evaluated, which limits the comprehensiveness of our toxicity profile. Additionally, the assessment of local control was unfeasible because of heterogeneity in post-treatment imaging and the inherent challenges in distinguishing tumor progression from treatment-related changes. Further prospective studies incorporating molecular profiling and standardized imaging criteria are warranted to refine patient selection and optimize salvage radiotherapy protocols.

## Conclusion

Salvage reirradiation with our protocol was feasible and safe, with outcomes comparable to those of prior studies. These results support its potential role as a treatment option for carefully selected patients with recurrent HGGs. However, given the retrospective design and small sample size, our findings should be interpreted with caution. Prospective biomarker-integrated trials and investigations of combination strategies with systemic or immunotherapies are warranted to further define the role of reirradiation in this setting.

## References

[1] Mizuhata M, Takamatsu S, Shibata S, Sakurai T, Minamikawa R, Yamazaki M, et al. Patterns of failure in glioblastoma multiforme following standard or short-course radiation and concurrent temozolomide. Jpn J Radiol. 2023;41:660–8. 10.1007/s11604-023-01386-2. 36648706 10.1007/s11604-023-01386-2

[2] Nishioka K, Takahashi S, Mori T, Uchinami Y, Yamaguchi S, Kinoshita M, et al. The need of radiotherapy optimization for glioblastomas considering immune responses. Jpn J Radiol. 2023;41:1062–71. 10.1007/s11604-023-01434-x. 37071249 10.1007/s11604-023-01434-x PMC10543135

[3] Murakami R, Hirai T, Nakamura H, Furusawa M, Nakaguchi Y, Uetani H, et al. Recurrence patterns of glioblastoma treated with postoperative radiation therapy: relationship between extent of resection and progression-free interval. Jpn J Radiol. 2012;30:193–7. 10.1007/s11604-011-0031-x. 22183828 10.1007/s11604-011-0031-x

[4] Kaul D, Pudlitz V, Böhmer D, Wust P, Budach V, Grün A. Reirradiation of high-grade gliomas: a retrospective analysis of 198 patients based on the Charité data set. Adv Radiat Oncol. 2020;5:959–64. 10.1016/j.adro.2020.06.005. 33083659 10.1016/j.adro.2020.06.005 PMC7557122

[5] Mayer R, Sminia P. Reirradiation tolerance of the human brain. Int J Radiat Oncol Biol Phys. 2008;70:1350–60. 10.1016/j.ijrobp.2007.08.015. 18037587 10.1016/j.ijrobp.2007.08.015

[6] Minniti G, Niyazi M, Alongi F, Navarria P, Belka C. Current status and recent advances in reirradiation of glioblastoma *[Review]*. Radiat Oncol. 2021;16:36. 10.1186/s13014-021-01767-9. 33602305 10.1186/s13014-021-01767-9 PMC7890828

[7] Gigliotti MJ, Hasan S, Karlovits SM, Ranjan T, Wegner RE. Re-irradiation with stereotactic radiosurgery/radiotherapy for recurrent high-grade gliomas: improved survival in the modern era. Stereotact Funct Neurosurg. 2018;96:289–95. 10.1159/000493545. 30404102 10.1159/000493545

[8] Fogh SE, Andrews DW, Glass J, Curran W, Glass C, Champ C, et al. Hypofractionated stereotactic radiation therapy: an effective therapy for recurrent high-grade gliomas. J Clin Oncol. 2010;28:3048–53. 10.1200/JCO.2009.25.6941. 20479391 10.1200/JCO.2009.25.6941 PMC2982785

[9] Chen ATC, Serante AR, Ayres AS, Tonaki JO, Moreno RA, Shih H, et al. Prospective randomized Phase 2 trial of hypofractionated stereotactic radiation therapy of 25 Gy in 5 fractions compared with 35 Gy in 5 fractions in the reirradiation of recurrent glioblastoma. Int J Radiat Oncol Biol Phys. 2024;119:1122–32. 10.1016/j.ijrobp.2024.01.013. 38232937 10.1016/j.ijrobp.2024.01.013

